# A search-based geographic metadata curation pipeline to refine sequencing institution information and support public health

**DOI:** 10.3389/fpubh.2023.1254976

**Published:** 2023-11-14

**Authors:** Kun Zhao, Katie Farrell, Melchizedek Mashiku, Dawit Abay, Kevin Tang, M. Steven Oberste, Cara C. Burns

**Affiliations:** ^1^Division of Viral Diseases, National Center for Immunization and Respiratory Diseases, Centers for Disease Control and Prevention, Atlanta, GA, United States; ^2^Cherokee Nation Businesses, Contracting Agency to the Division of Viral Diseases, Centers for Disease Control and Prevention, Catoosa, OK, United States; ^3^Division of Scientific Resources, National Center for Emerging and Zoonotic Infectious Diseases, Centers for Disease Control and Prevention, Atlanta, GA, United States

**Keywords:** ChatGPT, cloud computing, geographic locations, disparity of global sequencing capability, poliovirus, surveillance, sequence read archive

## Abstract

**Background:**

The National Center for Biotechnology Information (NCBI) Sequence Read Archive (SRA) has amassed a vast reservoir of genetic data since its inception in 2007. These public data hold immense potential for supporting pathogen surveillance and control. However, the lack of standardized metadata and inconsistent submission practices in SRA may impede the data’s utility in public health.

**Methods:**

To address this issue, we introduce the Search-based Geographic Metadata Curation (SGMC) pipeline. SGMC utilized Python and web scraping to extract geographic data of sequencing institutions from NCBI SRA in the Cloud and its website. It then harnessed ChatGPT to refine the sequencing institution and location assignments. To illustrate the pipeline’s utility, we examined the geographic distribution of the sequencing institutions and their countries relevant to polio eradication and categorized them.

**Results:**

SGMC successfully identified 7,649 sequencing institutions and their global locations from a random selection of 2,321,044 SRA accessions. These institutions were distributed across 97 countries, with strong representation in the United States, the United Kingdom and China. However, there was a lack of data from African, Central Asian, and Central American countries, indicating potential disparities in sequencing capabilities. Comparison with manually curated data for U.S. institutions reveals SGMC’s accuracy rates of 94.8% for institutions, 93.1% for countries, and 74.5% for geographic coordinates.

**Conclusion:**

SGMC may represent a novel approach using a generative AI model to enhance geographic data (country and institution assignments) for large numbers of samples within SRA datasets. This information can be utilized to bolster public health endeavors.

## Introduction

Outbreak response and preparedness require the proactive collection of all available public health–related information. To this end, the National Center for Biotechnology Information (NCBI) Sequence Read Archive (SRA) is a public repository of DNA sequencing data (1), providing access to large amounts of genomic data generated from organisms representing all branches of life (e.g., human, animal, plant, and microbes), as well as metagenomic and environmental samples. These data can be used to study the underlying causes of diseases, develop new treatments, track the spread of infectious diseases, and support outbreak responses.

Critically, for any dataset within the SRA, the potential for reuse or re-analysis to obtain deeper public health insights depends on the quality of both the genetic data and the associated metadata. Metadata refers to descriptive information that includes sample, experimental and/or data processing details (2). Accurate and complete metadata are essential for enabling public health authorities to understand the context of genetic data and assess possible relevance to public health concerns.

The quality of SRA metadata is generally considered to be high, as the NCBI makes substantial efforts to ensure completeness, consistency, accuracy, and correct formatting. However, like all large datasets, the SRA may contain errors and inaccuracies (3). These errors may arise from human mistakes (e.g., typographical errors, incorrect entries, missing information), technical issues (e.g., faulty data submission, transfer, or processing due to issues with the software, hardware, or network), and/or lack of standardization (e.g., expanded generation of user-defined properties and infrequent use of controlled vocabularies during data submission over time) (4, 5). As a result, SRA users may encounter absent or erroneous data across categories, including missing fields from data sources in the cloud, unclear synonyms, spelling variants, and heterogeneous sample data specification (2). These potential errors place the burden of responsibility on data users to carefully assess metadata quality and use the associated genomic data appropriately for public health related situations.

A potential gap within SRA datasets that may limit their application for public health research relates to the geographic location of the sequencing institutions from which the data were obtained. This information may not be readily available for every SRA sample but could be relevant to public health for several reasons. For example, sequencing institutions located in areas with a high burden of infectious diseases can play an important role in disease surveillance and outbreak investigations by quickly generating high-quality genomic data. Additionally, the availability of sequencing institutions located in a particular region can impact access to technology and resources for local researchers and public health practitioners. Moreover, sequencing institutions in a specific geographic region may have expertise in the types of diseases and pathogens prevalent in that area, with potential to inform the design and implementation of public health interventions, while ensuring they are culturally sensitive and ethically implemented. Lastly, the identification of laboratories with specific pathogens can support containment and inventory activities. For example, in the case of polio eradication, accurate identification of sequencing institutions that may possess potential polio infectious materials significantly enhances our ability to track and monitor the presence and transmission of the disease. Furthermore, such capability extends to scenarios where institutions unknowingly hold samples containing select agents or other high consequence pathogens; prompting further investigation by national authorities on containment.

To improve the usability of the imperfect SRA metadata for public health use, we developed a Search-based Geographic Metadata Curation (SGMC) pipeline that utilizes cloud technology coupled with ChatGPT. Newly emerging generative Artificial Intelligence (AI) technologies, like ChatGPT, are revolutionizing various fields, including public health research (6–8). These systems employ natural language processing and machine learning techniques to generate human-like text. We then applied this tool to identify the geographic location for 2,321,044 randomly selected and non-redundant samples in the SRA. Finally, we illustrate how SGMC can be used to uncover regional disparities in sequencing capability and support polio containment efforts. Although several strategies for curating existing metadata are currently being intensively studied (2, 5, 9, 10), to our knowledge, this is the first work to (1) determine the geographic locations of sequencing institutions in the SRA via cloud and ChatGPT, (2) apply the curated location information to elucidate disparities in global sequencing capability, and (3) highlight the potential application of SRA data for polio containment. Ultimately, we hope that this work will inspire further efforts to develop tools for enabling the use of publicly available genetic databases for improving human health.

## Methods

**Figure 1:**
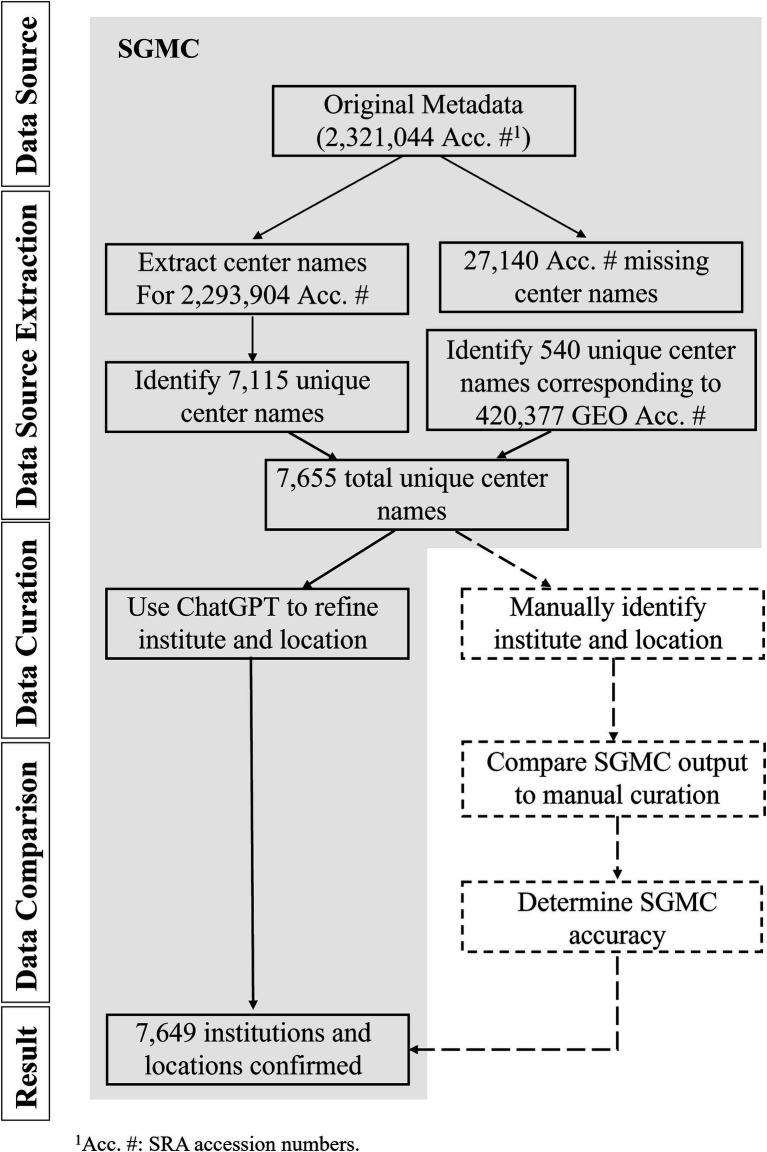
Visual representation of the Search-based Geographic Metadata Curation (SGMC) pipeline and results from the dataset analyzed in this study. The components of the SGMC pipeline were highlighted with a light gray shade, while additional analyses were represented through dotted lines and boxes.

To develop a computational tool for identifying the geographic location of any randomly selected SRA dataset, we constructed the SGMC pipeline using the SRA in the AWS cloud via Athena as of April, 2023 (11), Python v3.10.6, ChatGPT GPT3.5-turbo (12) and R v4.0.4 software. Our general workflow is described below and in Figure 1, and the source code can be found at https://github.com/CDCgov/PASS/tree/master/SGMC. This activity was reviewed by CDC, deemed not research, not involving human subjects, and was conducted consistent with applicable federal law and CDC policy.^1^

### Dataset description

Our dataset included 2,321,044 metadata entries with unique accession numbers randomly sampled from the public SRA database. A list of all accession numbers for the data used in this study can be found on GitHub. The data were checked for redundancy, and none was detected. Each metadata entry associated with a genetic dataset is broken up into attribute–value pairs, where the attribute specifies the metadata type (e.g., center name, sequencing technology, release dates), and the values are the corresponding metadata associated with that type. In this study, the determination of a sequencing institution is based on the value of the “center name” attribute that corresponds to a specific accession number found in the SRA metadata.

### Pipeline

Our pipeline is a Python-based information-retrieval application that includes four main steps: (1) data retrieval, (2) web scraping, (3) institution identification, and (4) location identification.

### Data retrieval via AWS cloud API

The Python script retrieves data from the NCBI SRA, which is accessed from the AWS via Athena. NCBI SRA metadata are available at s3:/sra-pub-metadata-us-east-1. Athena then uses the AWS Glue (crawler) service to generate tables from the metadata, allowing us to collect “center names” (CN; i.e., the possible institution where the sequencing was performed) for each accession number provided in a query.

### Web scraping

A web scraping method was implemented to obtain additional information from the NCBI webpage. During this step, three publicly available Python libraries (i.e., requests, BeautifulSoup, and pandas) and accession numbers (as unique keys) are used to fetch information in the “submitted by” (SB), “BioSample submission” (BS), and “BioProject submission” (BP) fields for each SRA accession. This process produces HTML-formatted data that were unavailable in the AWS Athena SRA metadata. An advantage of using web scraping is that it can extract information that may not be directly available in the SRA metadata but is available through NCBI’s website. Users can choose their favorite tool to retrieve additional information regarding the sequencing lab and are not required to use the web scraping methods in the pipeline.

### Prompt engineering for large language models

ChatGPT (i.e., OpenAI GPT-3.5) with default parameters was then used as an automated process to assign institution name, country, and geographic coordinates (latitude and longitude). Specifically, we input the extracted CN, SB, BS, and BP data related to each accession number into the ChatGPT model. The SGMC pipeline automatically generated and posed questions to ChatGPT. These questions not only aimed to discern the institution name, country, and geographic coordinates from these data but also included filtering and constraining instructions. Recognizing the presence of common occurrence filler words and abbreviations (e.g., generic database names rather than submitter’s names) in the database, we instructed ChatGPT to manage these and to seek alternative answers by filtering through other provided data. This ensured a more accurate institution name assignment. The AI model’s reliability was further bolstered using a retry mechanism, which would attempt up to three times with 60-s intervals if an initial request failed. The AI’s responses were appended to a result list, and upon reaching a partition size of 1,577 rows, data were saved to a file. Decisions around partition size and retry functions were driven by factors such as network connection, memory, and system performance.

An example of location determination that requires the sophistication of ChatGPT is the commonly used abbreviation “CAU,” which could be assumed to refer to Clark Atlanta University in Georgia, US. However, in the SRA metadata for accession SRR11871784, “CAU” refers to China Agricultural University in Beijing, China. Instead of solely relying on “CAU” to determine the location, it’s straightforward to instruct ChatGPT to utilize additional BioSample metadata associated with the accession. This metadata may include the details such as submitter’s name, the full name of the institute, or partial information related to geographic location, in the CN, SB, BS, and BP fields.

### Data analysis and accuracy evaluation

Output from SGMC (i.e., institute name, country, and geographic coordinates) was compared to results from manual curation of the data submitted to the NCBI from the United States (US), performed by subject matter experts (SMEs) at the Centers for Disease Control and Prevention (CDC), as the gold standard. Four distinct descriptors were assigned to each pairwise comparison of institute, country, and coordinates from SGMC and SMEs. “Concordant” (Table 1) was defined as the situation in which SGMC identified the same institute, country, or coordinates as the manual curation process. Comparisons for which the output was not the same were labeled “Discordant.” If SGMC was unable to identify the institute, country, or coordinates, the SGMC output was labeled “More Information Needed.” The label “collaboration” was applied in situations where collaboration across institutes was observed during the SRA submission process, and SGMC identified one of the collaborating institutes that was not chosen by manual curation.

**Table 1:** Accuracy analysis comparing SGMC results with human curation for institutions located in the United States.

	Concordant^a^	Discordant^b^	More information needed^c^	Collaboration^d^
Institution name	614 (94.8%)	0 (0%)	0 (0%)	34 (5.2%)
Country	603 (93.1%)	3 (0.5%)	37 (5.7%)	5 (0.8%)
Geographic coordinates	483 (74.5)	34 (5.2%)	124 (19.1%)	7 (1.1%)

The number outside the parentheses in each table cell represents the entry count within that category, while the number within the parentheses represents the percentage of that count with respect to the total count in that row. For example, 614 (94.8%) means that out of 648 institutions, SGMC identified 614 institutes that are the same as those identified through manual curation.

a Concordant is defined as the situation in which SGMC identified the same institute, country, or coordinates as manual curation.

b Discordant indicates disagreement between SGMC and manual curation.

c If SGMC was unable to identify the institute, country, or coordinates, the output was grouped in the “More Information Needed” category.

d Collaboration describes the situation in which multiple institutes collaborated during the SRA submission process, and SGMC identified a collaborating institute that was not chosen by manual curation.

Subgroups or multiple projects from a particular institute were merged under a single institute name. For example, CDC/Division of Healthcare Quality Promotion, CDC/Division of Bacterial Diseases, CDC/Pathogen Discovery and Detection Team, and CDC/National Center for HIV, Viral Hepatitis, STD, and TB Prevention were combined as CDC. Accessions that could not be identified by SGMC (i.e., “More Information Needed”) or were assigned the institute NCBI, European Bioinformatics Institute (EBI) (13), or Gene Expression Omnibus (GEO) (14) were re-run with the prompt to not include any variations of NCBI, EBI, or GEO as the listed institute. R was used to create heatmaps for the United States and world to display the number of samples submitted by each U.S. state and each country.

## Results

### Dataset

A flow chart illustrating the extraction and analysis process for the metadata analyzed in this study is provided in Figure 1. The original dataset included 2,321,044 accession numbers. Of these, 27,140 were missing center names and were excluded from further analysis. Among 2,293,904 accession numbers with center names, accession numbers associated with identifying center name information were isolated, and identifying data were condensed to 7,115 unique center names. Additionally, 540 more unique center names were extracted from 420,377 GEO accessions, resulting in a total of 7,655 unique center names. SGMC next employed ChatGPT to enhance the accuracy of the sequencing institution, country of origin, and geographic coordinates. This process resulted in confirming 7,649 refined sequencing institutions with 6 unique center names unable to be identified by both ChatGPT and manual curation, leaving a total of 7,649 unique and identifiable centers.

### Mapping sequencing institutions worldwide and in the United States

**Figure 2:**
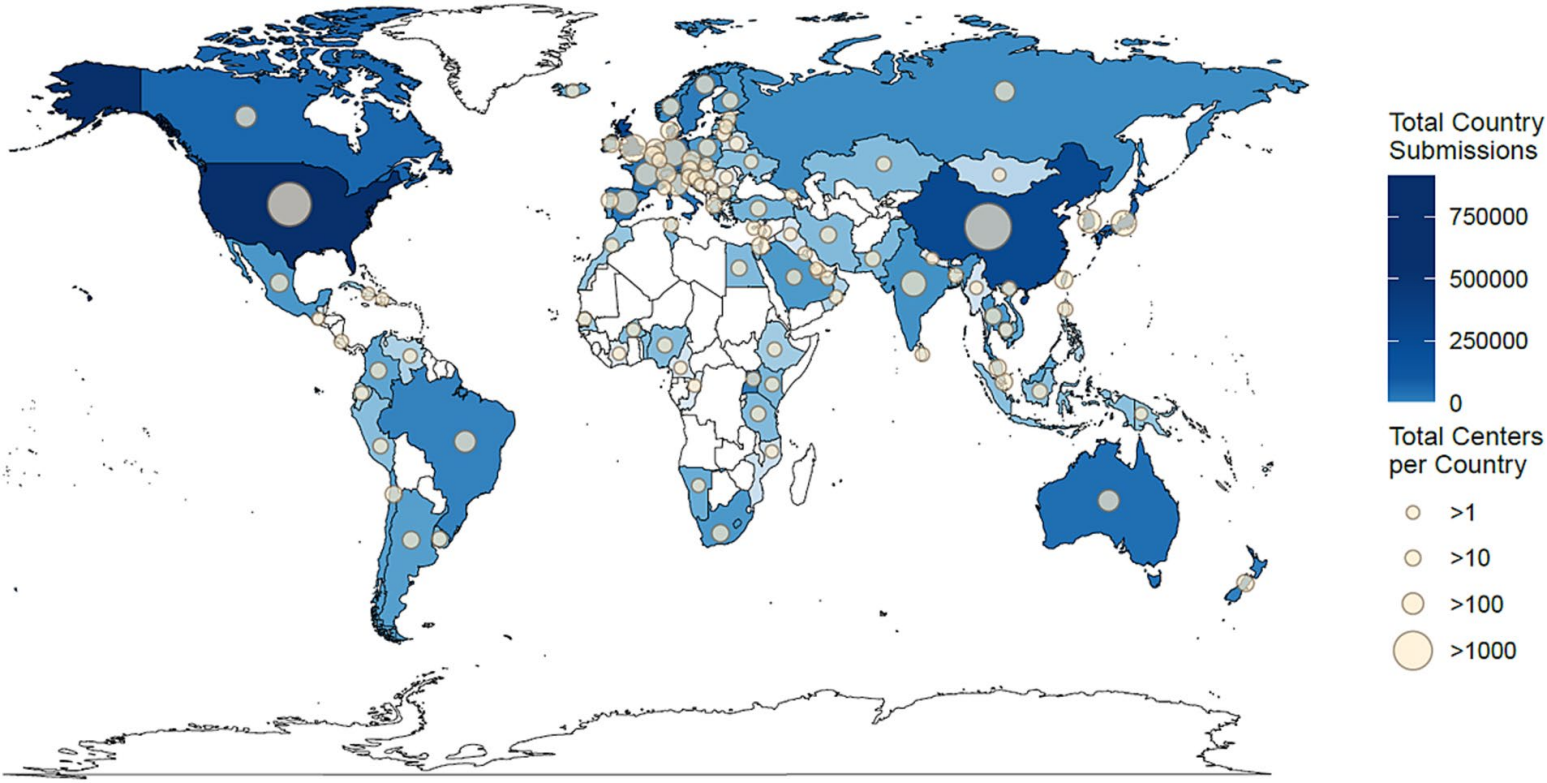
Geographical distribution of the 2,321,044 million samples that were sequenced or submitted globally by each country. Countries that submitted more sequencing data are shaded in darker blue, and those with fewer submissions are in lighter blue. Countries that did not contribute any samples are white. The number of submitted accessions ranges from zero to 1,001,358 (United States). The size of the circle on each country represents the number of institutions that contributed samples, with larger circles indicating a greater number of submitting institutions. Countries that did not have any institutions contribute to the data do not have a circle. The number of submitting institutions in each country ranges from zero to 2,043 (China).

SGMC was used to map identified geographic locations of sequencing institutions on the world and U.S. maps. Samples in this dataset were sequenced in 97 countries worldwide, with 42.2% (1,001,358) submitted from the United States (Figure 2). Following the United States, the United Kingdom and China generated the highest number of sequenced samples, 462,164 and 279,322, respectively. Almost all countries in North America, Western Europe, and South-East Asia submitted sequenced samples. However, data were lacking from many countries in Africa, Central Asia, and Central America. The country with the greatest number of centers submitting data to NCBI was China with 2,043 centers, followed by the United States with 1,525 and Germany with 445 (Figure 2).

**Figure 3:**
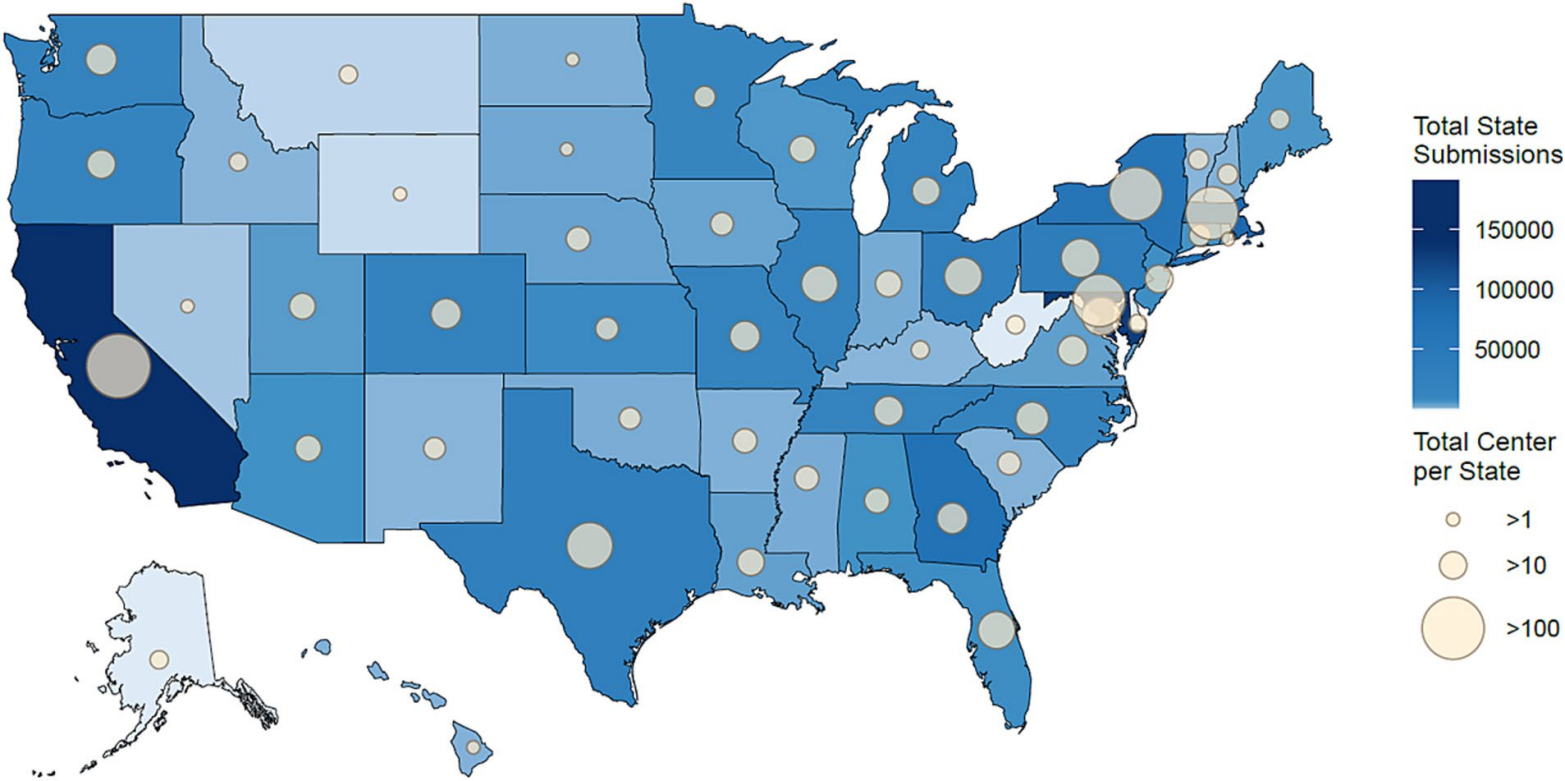
Geographical distribution of the 1,001,358 samples that were sequenced or submitted only in the United States by each state. States that submitted more sequencing data are shaded in darker blue, and those with fewer submissions are in lighter blue. The size of the circle on each state represents the number of institutions located in the state that contributed samples, with larger circles indicating a greater number of submitting institutions.

All 50 U.S. states and Washington, D.C. were represented among the submitted samples in this dataset. The number of institutions and submitted samples varied by state (Figure 3). California had the most data submitted to NCBI with 195,144 accessions. Maryland submitted the second most with 137,456 accessions, and Massachusetts was third, submitting 125,148 accessions. Similarly, California had the largest number of centers submitting data to NCBI with 102 centers, followed by Maryland and Massachusetts with 56 and 55 centers, respectively. In total, 36 states had 1–10 institutions submit to NCBI, 11 jurisdictions (10 states and Washington, D.C.) contained 11–50 submitting institutions, 3 states had 51–100 submitting institutions, and only 1 state had more than 100 institutes submit to NCBI. Overall, these findings highlight the potential for the SGMC pipeline to uncover sequencing disparities, both globally and within large countries such as the United States.

### Stratification of sequencing institutes

Furthermore, proof-of-concept analysis was performed to determine whether the data generated by our SMGC pipeline could be used for global public heath, focusing on polio eradication efforts. Sequencing plays a pivotal role in ongoing polio eradication efforts. Therefore, it becomes crucial to identify the geographical locations where genetic sequences are being generated, particularly if they are originating from labs outside the known Global Polio Laboratory Network (GPLN) sequencing facilities (15). By identifying these additional sources of sequencing data, we can gain valuable insights into the global landscape of sequencing efforts, which aids in enhancing surveillance, response strategies, and resource allocation to ensure the successful eradication of polio.

The 97 countries with 7,649 sequencing institutes in this dataset were stratified into the following categories: tropical countries^2^ (*n* = 39), lower-middle-income and low-income countries^3^ (*n* = 21), and polio-endemic, outbreak, and at-risk countries^4^ (*n* = 14). The remainder were not considered tropical, lower-middle-income and low-income, or at-risk for polio (Supplementary Figure S1). These results suggest that SGMC may be used to produce data that associate national disease status at a global scale.

### SGMC pipeline evaluations

All SGMC outputs regarding sequencing institutions located were manually curated results from SMEs but the accuracy was determined based on U.S. data due to better-defined geographic information, easier accessibility, and fewer language barriers. The accuracy was evaluated based on the institution names, countries, and geographic coordinates identified (Table 1). For institution name, no obvious error was observed in the SGMC output; 94.8% (614 out of 648) of SGMC institution identifications were consistent with human curation, whereas 5% of the identifications were inconsistent with SME curation due to the involvement of multiple institutes in the SRA submission process. Analysis of country identifications based on institution names revealed that 93.1% of the SGMC identifications were consistent with human curation. Only 0.5% of the country identifications from SGMC pipeline were inconsistent with manual curation due to the presence of multiple locations worldwide for some centers. In these cases, SGMC incorrectly identified the location after being provided the submitter’s name. In 5.7% of cases, SGMC was unable to identify any country and required additional information, and for 0.8% of cases, the country identification was the location for a collaborator who did not perform the sequencing. For geographic coordinates, the consistency rate decreased to 74.5%, and the inconsistency rate rose to 5.2%. 19.1% of geographic coordinates were not able to be identified, and 1.1% of coordinates was the location for a collaborator. This pattern is expected given that inferring accurate geographic coordinates is more difficult than only identifying an institution.

## Discussion

### Pipeline

Many automated or semi-automated methods aim to standardize metadata by clustering or mapping to ontologies (19); however, collecting precise metadata about locations remains a laborious and error-prone process (2, 4). In this study, SGMC employed a semi-automated technique that utilizes cloud and generative artificial intelligence approaches (i.e., ChatGPT) for the curation and update of geospatial metadata from the NCBI SRA. Our results show high consistency between SGMC results and human curation, suggesting the potential for SGMC to save human efforts, reduce errors, and enable scale-up of manual curation processes for improving the usability of public data.

By developing the SGMC pipeline, we hope to provide users with a flexible tool that can be readily customized to meet the needs of different users and studies. For example, the script could be modified to retrieve data from different sources or to use alternative natural language processing techniques [e.g., Google Bard (20, 21) and Fuzzy methods (22)] for data analysis. In addition, the output format of the extracted data could be modified to suit the user’s needs.

### Data quality

For an accurate understanding of the geographical distribution of a given event involving specimen sequencing, it is helpful if location metadata are accurate and up to date. Prior to this study, the accuracy and completeness of the SRA metadata with respect to sequencing submitters’ institutions were unclear. Moreover, not all sequencing centers routinely provide this information, and the quality and completeness of the metadata can vary depending on the experiment and the individual submitter. For example, the geographic location of sequencing centers submitting their samples to the SRA can be provided in various ways, such as through the name of the institution or the city and country where the sequencing center is located.

Of note, the 94% consistency observed between SGMC sequencing institution results and those obtained from manual curation suggests that the quality of SRA data for U.S. institution identification purposes is high. Consistent with this interpretation, we found that within our dataset, the rate of missing institution names for SRA accessions was very low (<2%).

### Public health implication for global sequencing capability

One immediate application of using the SGMC pipeline is to update understanding of the geographic distribution of sequencing centers that are available to share data with the public during a global health crisis. Unfortunately, the current dataset reveals an unequal distribution of sequencing technologies and resources across the world. The global burden of infectious disease is disproportionately carried by low- and middle-income countries, whereas advanced sequencing techniques for disease surveillance are primarily available in wealthier nations (23). Accordingly, some countries appeared to be overrepresented among the submitting sequencing centers identified in this study. Our findings suggest this global imbalance could be the result of differences in genomic capacity. Specifically, lack of sequencing capability (and diagnostic capacity in general) in low- and middle-income countries may contribute to their higher disease burden and poorer health outcomes.

### Public health implications related to infectious material containment and surveillance

Another immediate application of SGMC is in support of disease containment and surveillance. Using polio containment as an example, we note that the potential applications of the public sequencing archive outside the scope of the GPLN have been understudied (24, 25). Datasets within the SRA have the potential to provide additional information for strengthening polio surveillance and containment efforts outside the GPLN. For example, although most sequencing projects do not include poliovirus as a primary target, the incidental detection of poliovirus in any of those non-polio samples, e.g., by using metagenomic methods, can have a significant impact on public health. This is because identification of poliovirus in a polio-free area triggers additional surveillance efforts and an urgent outbreak response. Consequently, the identification of sequencing institutions is highly relevant to containment and surveillance activities. Our findings further suggest that use of the SRA for polio containment and inventory survey could be more beneficial in countries where more samples are sequenced. Importantly, containment efforts for other pathogens may also benefit from automated extraction of data in the NCBI SRA.

### Limitations

This work has several limitations. First, descriptive statistics of geographic information for sequencing institutions in the SRA were limited to those randomly selected by a specific date in April 2023. Given that the SRA database is continually subject to changes or updates, re-running the SGMC pipeline in the future may provide new insights. Second, it may not always be appropriate to infer the sequencing capability of a particular country or region based on the SRA database alone, since some researchers may submit their data to repositories not fully connected to the U.S. NCBI [e.g., Genome Sequence Archive in China (26)], or they may choose not to share the data at all. However, U.S. NCBI is part of the International Nucleotide Sequence Database Collaboration (INSDC) (27) alongside the DNA Data Bank of Japan (DDBJ) (28) and the European Nucleotide Archive (29). Any data submitted to the three databases is accessible from the others. Thus, we expect that trends and patterns reported in this work reflect general sequencing capabilities on a global scale (30). Third, the non-deterministic nature of LLM outputs and the non-static nature of public models remain in practice. We are not concerned in this preliminary report because the prompt question was specifically asked in a way that minimizes variation or possible answers to the questions. Sharing code on GitHub is intended to encourage users to adapt the code to their own understanding of non-static datasets. Finally, the SGMC approach is semi-automated, as manual curation is still required to confirm results. For example, sequencing collaborators usually involved laboratories where sample preparation occurred before sending the sample to a sequencing institute. In cases where only one location can be used to track the geographic origin of the sequencing, additional efforts to gather information from other sources may be necessary, and the final decision regarding which location to use is subject to human determination. Regardless, we anticipate use of cloud-based databases and generative artificial intelligence tools, such as ChatGPT, for improved data management and productivity in future public health work. However, fully grasping the potential risks and limitations of these approaches is necessary to acquire a deeper and more precise understanding of the data and how they may be applied to the public health domain.

In summary, SGMC may represent a novel approach that employs a generative AI model to enhance geographic data (country and institution assignments) for large numbers of samples within SRA datasets. This information can be utilized to bolster public health endeavors.

## Data availability statement

The datasets and code presented in this study can be found in online repositories. The names of the repository/repositories and accession number(s) can be found at: https://github.com/CDCgov/PASS/tree/master/SGMC.

## Author contributions

KZ: Conceptualization, Data curation, Formal analysis, Funding acquisition, Investigation, Methodology, Software, Writing – original draft, Writing – review & editing. KF: Data curation, Formal analysis, Software, Visualization, Writing – original draft. MM: Data curation, Formal analysis, Investigation, Methodology, Software, Writing – original draft. DA: Data curation, Methodology, Software, Writing – review & editing. KT: Investigation, Writing – review & editing. MO: Funding acquisition, Project administration, Resources, Writing – review & editing. CB: Conceptualization, Funding acquisition, Investigation, Project administration, Resources, Writing – review & editing.

## Footnotes

^1^See e.g., 45 C.F.R. part 46.102(l)(2), 21 C.F.R. part 56; 42 U.S.C. §241(d); 5 U.S.C. §552a; 44 U.S.C. §3501 et seq.

^2^Tropical and non-tropical countries were defined in Phalan et al. (16).

^3^High-income, upper-middle-income, lower-middle income and low-income countries were defined by United Nations (17) and according to World Bank’s Country classification by income (https://datahelpdesk.worldbank.org/knowledgebase/articles/906519).

^4^Polio-endemic, outbreak, and at-risk countries were defined in GPEI (18).

## Supplementary material

Supplemental Figure 1 Venn diagram showing number of sequencing countries and their institutes based on country income classification (lower to middle income), category related to polio-risk assessment in 2023 (polio-endemic, outbreak, and at-risk), and climate region (tropical). The number outside the parentheses represents the number of countries within that category, whereas the number within the parentheses represents the number of institutions located in those countries. For example, in the plot, 5 (31) means that there are 31 institutions in five lower-middle income countries that are neither polio-outbreak or at-risk countries nor in tropical areas.
