# Supplementary figures and images for: A search-based geographic metadata curation pipeline to refine sequencing institution information and support public health
Source: Front Public Health. 2023 Nov 14;11:1254976. doi: 10.3389/fpubh.2023.1254976 (PMC10683794; doi:10.3389/fpubh.2023.1254976)

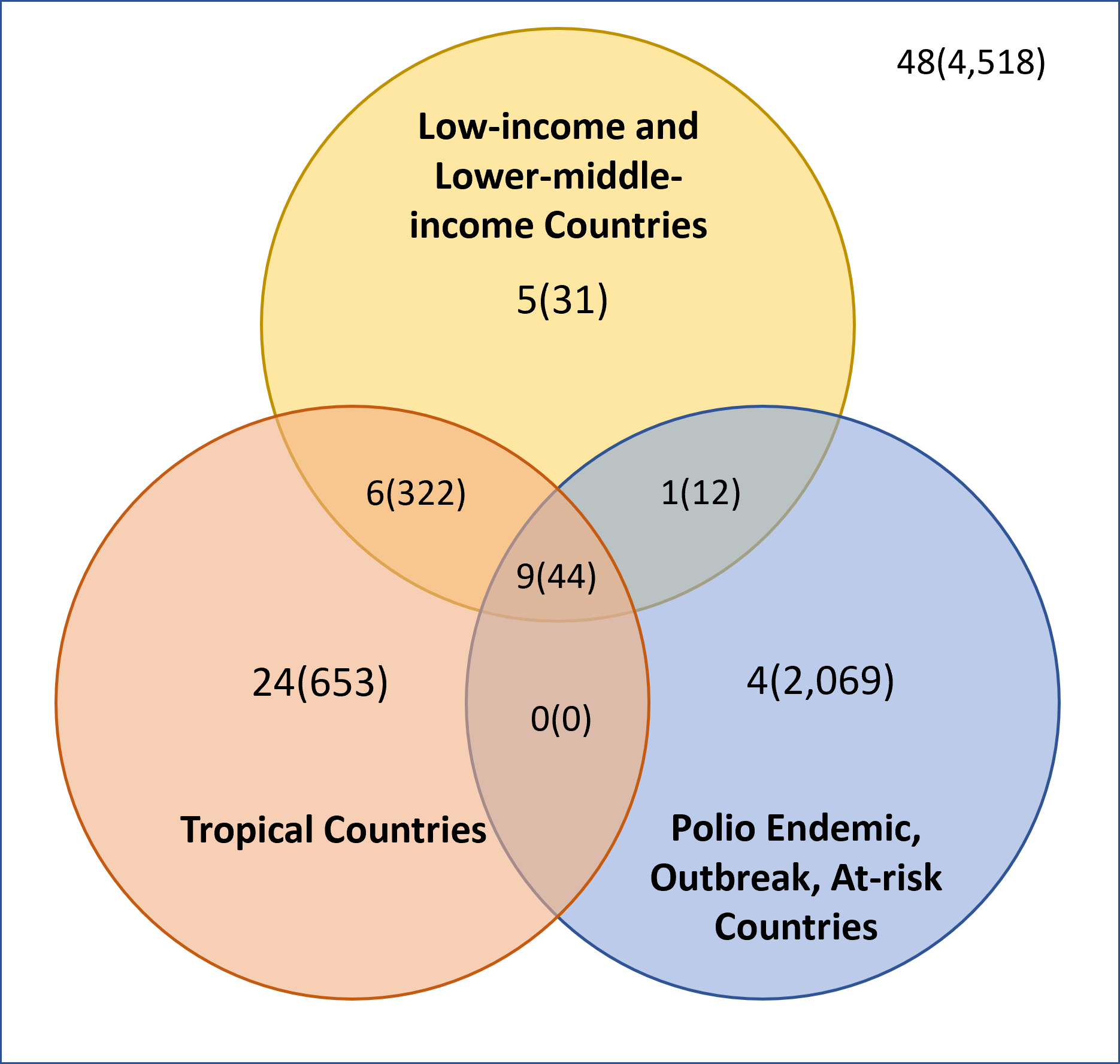

Supplement: Supplemental Figure 1 — Venn diagram showing number of sequencing countries and their institutes based on country income classification (lower to middle income), category related to polio-risk assessment in 2023 (polio-endemic, outbreak, and at-risk), and climate region (tropical). The number outside the parentheses represents the number of countries within that category, whereas the number within the parentheses represents the number of institutions located in those countries. For example, in the plot, 5 (31) means that there are 31 institutions in five lower-middle income countries that are neither polio-outbreak or at-risk countries nor in tropical areas. [file Image_1.TIF]
